# Radiation-Driven Migration: The Case of Minamisoma City, Fukushima, Japan, after the Fukushima Nuclear Accident

**DOI:** 10.3390/ijerph110909286

**Published:** 2014-09-09

**Authors:** Hui Zhang, Wanglin Yan, Akihiro Oba, Wei Zhang

**Affiliations:** 1 School of Urban Culture, South China Normal University, Nanhai, Foshan 528225, China; 2 Graduate School of Media and Governance, Keio University, 5322 Endo Fujisawa, Kanagawa 252-0882, Japan; E-Mails: yan@sfc.keio.ac.jp (W.Y.); perry@sfc.keio.ac.jp (A.O.); 3 College of Public Administration, Huazhong University of Science and Technology, Wuhan 430074, China; E-Mail: weizhangscu@gmail.com

**Keywords:** radiation contamination, Fukushima nuclear accident, migrant, aging, depopulation, recovery, Minamisoma

## Abstract

The emigration of residents following the Fukushima nuclear accident has resulted in aging and depopulation problems in radiation-contaminated areas. The recovery of affected areas, and even those areas with low radioactive pollution levels, is still heavily affected by this problem. This slow recovery consequently affects immigration patterns. This review aims to present possible factors that have contributed to this dilemma. We first present an overview of the evacuation protocol that was administered in the study area following the Fukushima accident. We then analyze characteristics of the subsequent exodus by comparing population data for both before and after the accident. Based on the findings of existing literature, we identify three causes of emigration: (1) The health risks of living in a low radiation zone are still unknown; (2) The post-disaster psychological disturbance and distrust of government information promotes the emigration of evacuees; (3) an absence of economic vitality and of a leading industry renders the area less attractive to individuals residing outside of the city. Further research is needed on this issue, especially with respect to countermeasures for addressing this problem.

## 1. Introduction

The Great Eastern Japan Earthquake and subsequent tsunami that occurred in March 2011 resulted in a nuclear accident at the Fukushima Daiichi Nuclear Power Plant (FDNPP), which is affiliated with the Tokyo Electric Power Company (TEPCO). The accident was deemed to be one of the most devastating nuclear disasters in human history. Shortly after the earthquake, a tsunami inundated the power plant, resulting in a power failure and the breakdown of the reactor cooling system. The overheated reactors started to melt down, causing a hydrogen-air explosion and releasing a large volume of radioactive material, including 1.6 × 10^17^ Bq of ^131^I and 1.5 × 10^16^ Bq of ^137^Cs [1]. Contaminating an area larger than 1700 km^2^ [2] and forcing the evacuation of 146,520 people from Fukushima Prefecture [3], the nuclear explosion was given a Level 7 rating on the International Nuclear and Radiological Event Scale (INES) by the International atomic Energy Agency (IAEA) [4], thus rendering the accident the most severe case since the Chernobyl accident. 

Three years after the triple disaster (earthquake, tsunami and nuclear explosion) struck Japan, the affected area is undergoing recovery. However, reconstruction efforts in the radiation-contaminated area are not progressing as quickly as those in areas that were only affected by the earthquake and tsunami. The first obstacle to reconstruction in this area concerns the outmigration of residents and aging of the population [5]. Evacuees, and especially young people, are reluctant to return to areas surrounding the FDNPP, and even to regions that have been declared safe, with low radioactivity levels [6]. Minamisoma city, located 14–38 km north of FDNPP, is one of the cities that was most severely affected by the nuclear explosion. Forty-two percent of the evacuees from Fukushima Prefecture were residents of this city [7]. Three years after the disaster, the city’s population only reaches 68.2% of the pre-disaster population size, 32.88% of which is composed of senior citizens of over 65 years of age. The Japanese Nuclear Emergency Response Headquarters (NERHQ) has lifted the evacuation call based on radiation monitoring results, declaring that most areas in Minamisoma are safe for residence, and a number of scientific institutions have also concluded that radiation levels in regions where evacuation calls have been lifted are within safe limits [8,9]. Despite this, there has not been a dramatic increase in the willingness to return. Population decline and aging accompanied by an acute labor force shortage and lack of economic vitality have significantly hindered post-disaster recovery while increasing the vulnerability of these communities. 

Prior to the Fukushima accident, only the Chernobyl accident, which occurred in the Soviet Union (1986), had reached a Level 7 INES rating [2]. More information is thus needed to develop a more comprehensive understanding of the state of the radiation-affected area. The post-disaster Minamisoma recovery plan provides directives on appropriate responses to future nuclear accidents. While numerous research reports have been produced on the Fukushima accident, the majority focuses on radiation effects rather than economic and social aspects. Through a case study of the demographic situation in Minamisoma, the present study, in view of existing literature and reports, discusses demographic problems afflicting radiation-affected areas. The paper begins with a brief overview of the evacuation protocol that was followed in the study area. The demographic situation in Minamisoma and its impact on recovery in the area is then analyzed. Finally, major factors that are restricting immigration to the area are identified. Relevant health influence, psychological resilience and economic frustration are found to be the main factors that are contributing to this problem.

## 2. The Study Area: Evacuation Plan and Restoration Efforts

The paper focuses on the demographic change in the areas stricken by the Fukushima nuclear accident and the possible affecting factors for the change. The selection of Minomisoma City as a case study is based on the following reasons: (1) Minomisoma is highly representative of seriously affected areas. Within the radius of 60 km around FDNPP, Minomisoma is regarded as one of the most seriously affected areas in the accident. Radionuclides of different concentrations have been detected in the air, the soil, and the rivers after the accident [6]. (2) Minomisoma faces both evacuation and return problems. As high as 42% of Fukushima evacuees come from Minamisoma, which means the evacuation and return problem is quite concentrated in this area [7]. After the decontamination and the re-measurement of radiation, NERHQ claimed the safety of Minomisoma residence and gradually lifted the residence restrictions in most parts of this city, which means return is possible and feasible.

**Figure 1:**
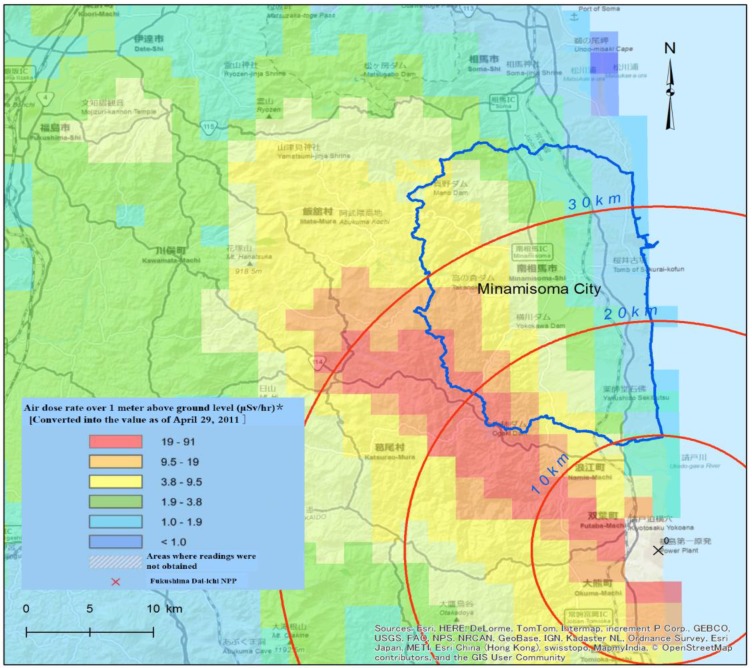
Exclusive Radiation Zones around FDNPP and Location of Study Area [10,11].

*The does rate was obtained in µSv/hr (equivalent dose), which can be converted to mSv/yr using the formula: 1 µSv/hr = 8.76 mSv/yr.

The discussions in this paper are likely to apply to most cities affected by Fukushima nuclear accident except some restricted areas such as Namie Town, Futaba Town, Okuma Town, Tomioka Town, Naraha Town [10]. Residence is forbidden for a considerable period in these restricted areas, which means there is no return problem for the time being. 

Minamisoma City was founded on 1st January 2006 through a merger between Haramachi City, Kashima Town and Odaka Town. In February of 2011, the city registered a population of 71,561. Located in the northern region of Fukushima Prefecture and facing the Atlantic Ocean to the east, Minamisoma covers a total area of 398.50 km^2^, 55% of which is covered by mountains and forests. Prior to the disaster, agriculture and manufacturing acted as the leading industries in the area [12].

**Figure 2:**
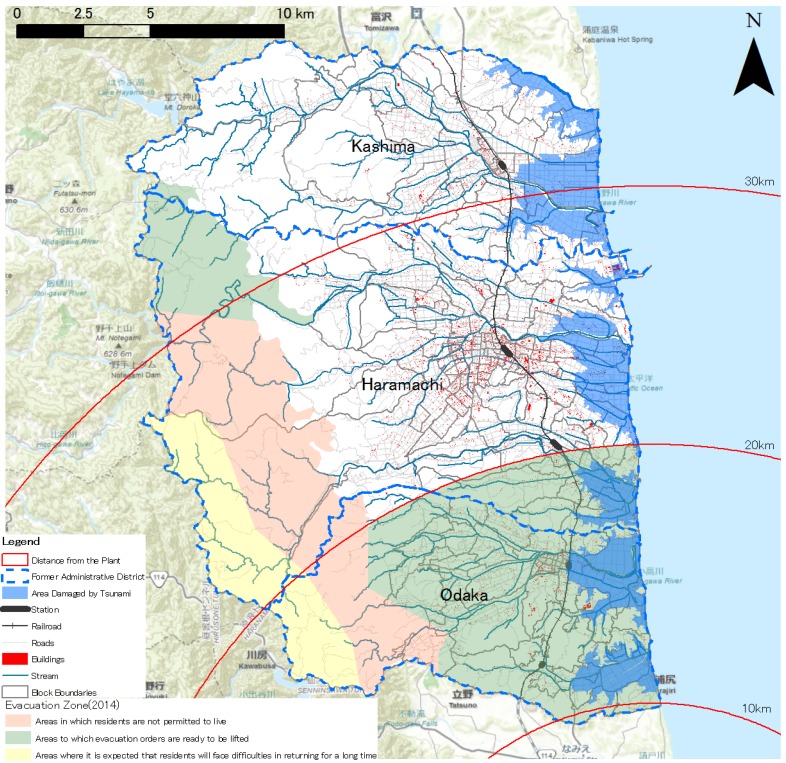
The Evacuation Zone in Minamisoma Established after the Fukushima Accident [13,14].

On 11 March 2011, The Fukushima nuclear accident happened near Minamisoma City. In response, the NERHQ created a 20 km-radius exclusion zone around the FDNPP and ordered residents of the area to evacuate (Figure 1). On 14th March, Unit 3 of the FDNPP reactor exploded, and fuel rods in Unit 2 were fully exposed due to inadequate cooling [1,15]. As a consequence of the nuclear explosion, relief supplies could not be transported into the city, leaving citizens in a severe shortage of basic necessities. Shortages of food and petrol spurred fears and chaos, and residents began to evacuate the area. Unclear evacuation instructions caused numerous residents to flee to the northwestern zone where radiation levels were even higher [16].

On 15th March, FDNPP Unit 4 began to melt down. On the same day, the NERHQ issued a “stay-in-house” order to residents situated within a 20–30 km radius around the FDNPP (Figure 1) [17]. The municipal government began to evacuate residents in groups by bus. Since then, most residents in the city have found shelter on their own accord or with government assistance. One month after the accident, the population of Minamisoma dropped drastically from 71,561 to approximately 10,000 [5]. On 16th March, the NERHQ issued directive recommending that residents under the age of 40 remain as far away from the FDNPP site as possible and began to distribute iodine tablets to the public [2,17]. However, few residents in Minamisoma took the iodine tablets because the evacuation had already been completed [18]. On 21st April, a 20 km exclusion zone around the FDNPP and an area of 107 km^2^ in Minamisoma, including the district of Haramachi and the southern section of Odaka, were cordoned off (Figure 2). All residents in the exclusion zone were forced to evacuate. A 20–30 km-radius area was also demarcated as the Planned Evacuation Zone, within which residents were required to remain at home as much as possible and remain prepared to leave if conditions deteriorated [17]. A 181-km^2^ area that included northern Odaka and southern Kashima was situated within this zone. As a consequence of the establishment of distinct radiation zones, residents who fled in dread began to come back, and number of residents in the city rebounded to 25,000 in April of 2011 [19].

Soon after the Fukushima disaster, the Japanese government began to monitor radiation levels and decontaminate radiation-affected areas. Features that are monitored include (1) spatial radioactivity, (2) cultivated land and soil, (3) rivers and seas, (4) drinking water, milk, vegetables, fruits, meat and fish, and (5) exported manufactured goods [19,20,21]. Because nuclear radiation is considerably more damaging to children and adolescents than adults, the emphasis of monitoring has been placed on schools, roads that lead to schools and parks. In July of 2011, the government of Minamisoma formulated *The Strategy for the Decontamination of Radioactive Substances in Minamisoma*. The strategy strives to decontaminate residential areas to an annual cumulative radiation dose of over 5 mSv and over 5000 Bq per kilogram of soil for cultivated land. The initial goal is to reduce radioactivity levels by 60% within three years [22]. In July of 2012, the Environment Administration formulated *The Special Decontamination* *Area Plan*, which aims to decontaminate areas within the 20 km radius area around the FDNPP that receive annual cumulative doses higher than 20 mSv [23]. By 15th April 2014, approximately 19% of the residential areas were decontaminated to this level [24].

On 7th August 2013, the NERHQ rezoned the city to include difficult-to-return areas, restricted residency areas, and areas that no longer need to remain evacuated based on radioactivity levels measured following cleanup efforts (Figure 2). In difficult-to-return areas, radiation doses are higher than 50 mSv/yr, and residents are unable to return for a long period of time. In restricted residential areas, doses range between 20 and 50 mSv/yr, and while residents may enter these areas, they may not stay overnight. In areas where evacuation requirements have been lifted, doses fall below 20 mSv/yr [25]. In Minamisoma, only a small area to the northwest of the FDNPP has been categorized under difficult-to-return or restricted residency area categories, and thus most of the other areas are now reopened to residents due to low-level radioactivity in these areas.

## 3. The Post-Disaster Population Situation and Its Effects

### 3.1. Post-Disaster Population Data for Minamisoma

Minamisoma registered a population of 71,561 people prior to the disaster [12]. Although some evacuees returned home after the NERHQ lifted evacuation requirements for most areas of the city, the rate of return has been very low. In March of 2014 (three years after the disaster), the post-disaster population size of Minamisoma reached only 66% of the pre-disaster population levels.

**Figure 3:**
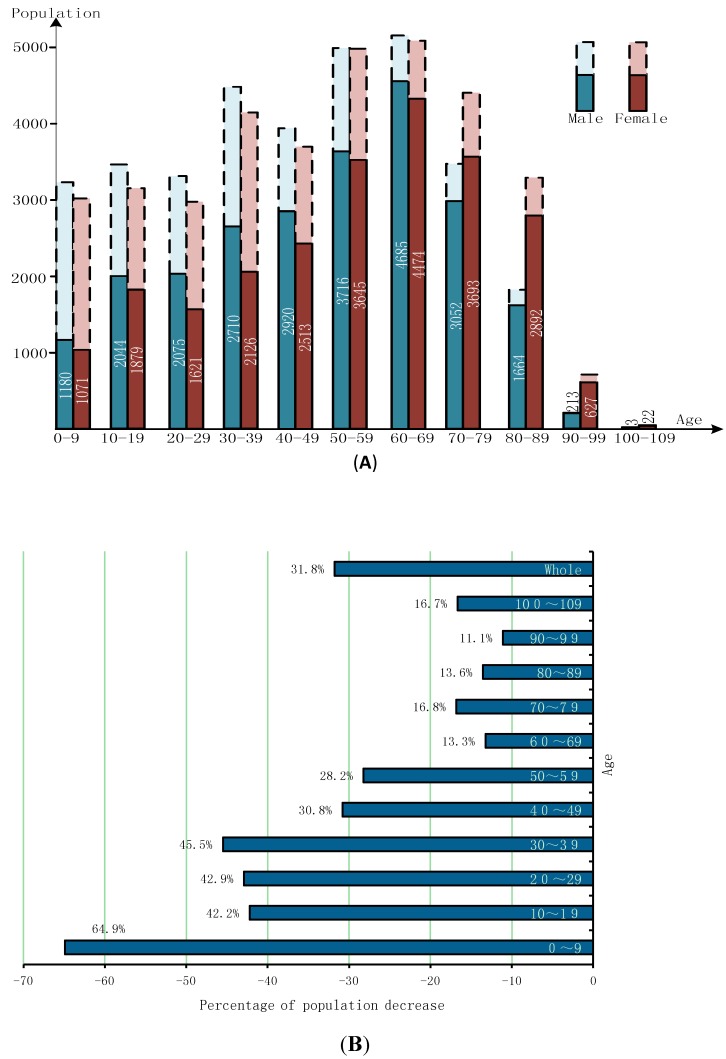
**(A)** Population Remaining in Minamisoma after the disaster by Gender and Age. **(B)** Percentage of Population Decrease by Age (comparison between 10th March 2014 and 11th March 2011) Data Source: Statistics Department of Information Policy, Division of General Affairs Office of Minamisoma.

Residents that have not returned to Minamisoma are predominantly young adolescents under the age of 18 and adult women. As shown in Figure 3, younger evacuees are the least likely to return. For example, the current population of infants and children aged 0–9 is only 35% of what it was prior to the disaster. Slightly more males have returned than females, and this trend is most prominent among individuals aged 20–39, among whom the proportion of male returnees is 9.2% larger than that of female returnees. The low proportion of child and female returnees may be attributed to the fact that children often remain with mothers, who opted to emigrate. The population of over 40 years of age forms the largest cohort of residents that returned to the city after the disaster. Among senior residents older than 65, 86% have returned. In contrast, 37% of the population aged under 40 and approximately 22% of the population aged between 40 and 59 moved to other cities. 

**Figure 4:**
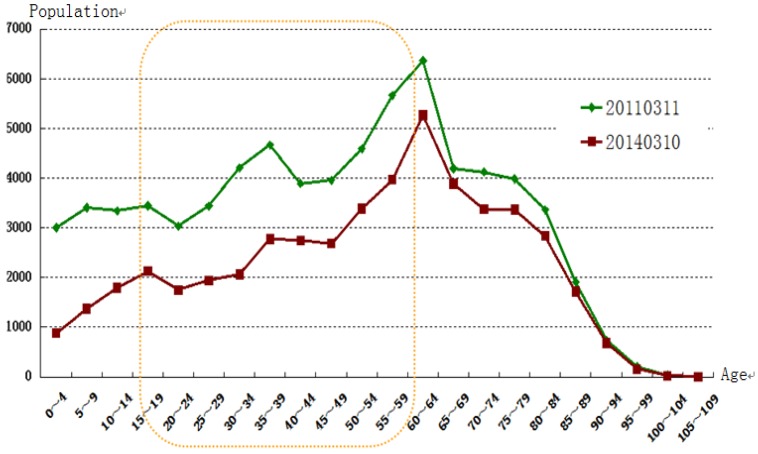
Comparison between Labor Force Populations in Minamisoma before and after the Fukushima Accident. Source: Statistics Department of Information, Policy Division of General Affairs Office of Minamisoma.

With the young population emigrating and the elderly staying behind, disaster-stricken areas are characterized by aging populations and dramatic labor force decline. The percentage of seniors over the age of 65 residing in Minamisoma increased from 25.92% on 11th March 2011 to 32.88% on 10th March 2014. In contrast, among evacuees that have migrated to other cities, only 16.66% are elderly residents. As shown in Figure 4, in stark contrast to the 33% labor force decrease in Minamisoma, more than 83% of evacuees now residing in other cities are younger than 65.

### 3.2. The Effects of Evacuation and Demographic Change

A quickly shrinking and aging population has far-reaching impacts on all aspects of society, *i.e.*, economy, politics and culture [26]. Though population decline may reduce human impacts on the environment and improve living conditions to some extent, its adverse effects on disaster-stricken areas may far outweigh its benefits. After the Fukushima accident, the population of Minamisoma declined to 66% of that prior to the accident, and the average age of residents increased by 14 years, a level that was expected to reach by 2025 [5]. The Fukushima Accident has left Minamisoma with no other choice but to confront this problem head on and make necessary policy adjustments. In the following section, we discuss the possible impacts of population aging and decline on areas affected by nuclear accident—the long-term effects and features of community vulnerability and weakened resilience.

Extensive research has been conducted on the impact of population aging on disaster recovery and community vulnerability. Though it was previously believed that the elderly are more psychologically resilient to disasters, these individuals may also need to invest more time and effort into disaster recovery due to their physical vulnerability [27,28,29]. A proportional increase in the elderly population thus renders a community as a whole more vulnerable and less resilient to disaster [30]. Elderly take more health risks during the first stage after the accident—evacuation time. First, the health risks may arise in the course of relocation. Due to the lack of clear evacuation instructions and the reluctance of some local governments to accept evacuees, some evacuees have to suffer from several transfers within a few months after the accident. During this process, the health conditions of many aged inpatients deteriorate continuously because of the lack of sound and consistent medical care. The death rate of the evacuated elderly inpatients soars up within the first three months after the accident. Although the rate declines notably afterward, it is still higher than the pre-disaster rate. From March to October 2011, 295 inpatients in evacuation zone died in the evacuation process, among whom 93% of which were seniors over 75 years in age. Of those, 40.9% died from pneumonia, which was believed to be caused by the low temperature and nutritional deficiency in the course of relocation [31]. Second, the health risks arising from living in the temporary housing for a long period of time. The water, power and heating supplies in temporary housings were often in short supply. In the meantime, it is hard for evacuees suffering from chronic diseases to get medication and special foods. Their symptoms may consequently worsen because regular check-ups and treatment are also unavailable. The hard life leads to a striking increase in number among the evacuees suffering from certain diseases, such as deep-vein thrombosis (DVT) and disuse syndrome [32]. Of the 305 disaster-related deaths that occurred in the year following the disaster, 298 of these individuals were older than 70 years of age, and this trend is growing more pronounced [5]. Moreover, the psychological well-being of evacuees is also a big concern. The change of environment, less social activities and the fear of nuclear radiation make evacuees vulnerable to such psychological diseases as post-traumatic stress disorder and major depressive disorders [33]. 

In the meantime, the number of seniors who need nursing care increased from 2761 in February of 2011 to 3380 in May of 2013, an increase of approximately 29%. However, 56% of the hospitals and clinics in Minomisoma were forced to shut down after the disaster, and the number of doctors and nurses on duty declined by 15% and 19%, respectively [6]. Faced with an apparent contradiction between a surging demand for medical services and a sharp decrease in medical resources, the Minomisoma government must prioritize restoring medical resources as part of its reconstruction budget, which may otherwise be dedicated to the restoration of other forms of infrastructure. For example, three years after the disaster, certain areas of Minamisoma are still insufficiently connected to public services, with no access to electricity, running water or public transport [24]. These inefficiencies and inadequacies disrupt the normal operation of enterprises and the lives of residents and thus delay economic recovery.

Among the numerous resources required for reconstruction, a sufficiently large labor pool is the most fundamental. In Minamisoma, the recovery process has been hindered by the outflow of labor following the Fukushima accident [6]. The 33% reduction in the working-age population (aged 15–65) has left Minamisoma, causing such a severe shortage of labor that numerous new jobs created for reconstruction have not yet been filled [34]. The gap between the number of job vacancies and job seekers is widening. According to statistics, by April of 2013, there were 1.92 times more jobs than applicants in Minamisoma [6]. This widening gap has also occurred because in an aging society, the young and middle-aged population must dedicate more time to caring for senior family members and friends. This cohort of the labor force should be devoted to more productive activities. The repercussions of the severe shortage of labor are evident in all areas of recovery: decontamination progress is slow; service industries must limit business hours; industrial enterprise production cannot function at full capacity. In the meantime, the dramatic decline in the number of consumers in this area undermines incentives to invest in Minamisoma. In sum, the depopulation problem has decreased the economic vitality of this city, rendering the city no longer appealing to investors [26,34]. 

Though Minamisoma is not the only city to experience rapid depopulation and aging in the wake of disaster, it differs from other cases in that radiation contamination, apart from earthquake and tsunami effects, complicates the nature of post-disaster recovery. With radiation threats looming large in resident psyches, the shortage of labor has not been alleviated overtime and may not be assuaged for a long time to come.

## 4. Reasons for Emigration

### 4.1. Relevant Health Influence

It has been confirmed that a certain level of nuclear radiation increases exposed group’s susceptibility to all solid cancer and leukemia and causes greater harm to fetuses, children than adults [35,36,37,38,39]. UNSCEAR (United Nations Scientific Committee on the Effects of Atomic Radiation) reported that the absorbed effective dose is higher among the exposed group who live in the non-evacuate areas within Fukushima than residents who live in other areas of Japan [38,39]. In other words, provided there is no future remediation and protective measures, evacuees who return to the non-evacuate areas within Fukushima, may take more radiation-related health risk in the future, especially for the infants and children. The World Health Organization also noted that “with respect to Japan, this assessment estimates that the lifetime risk for some cancers may be somewhat elevated above baseline rates in certain age and sex groups that were in the areas most affected.” [8]. However, UNSCEAR also declared in the report, “the values of inferred risks are so small that in general no discernible radiation-related increase of overall cancer incidence would be expected among exposed members of the general public” [38]. Most other institutions and researchers also agree that radiation contaminants discharged from the Fukushima accident will have a negligible effect on the health of local residents [8,9,40]. For most residents of Fukushima, the radiation does would not increase the occurrence of cancer and other ailments [9]. A comprehensive survey conducted on 9619 Minamisoma residents in June of 2013 also confirmed that no internal radiocesium were found in 99% of children and in more than 90% of adults [41]. As the radioactive contaminants may be eliminated from the body by excrement and urine, the number of population with internal radiocesium would decrease every month, provided there is no fresh intake any more. However, some other long-lived radionuclides are bone-seekers that exhibit a very long residence time in the human body, e.g., Sr-90 and Pu, but they are severely understudied [42,43,44,45,46]. 

As the uncertainty of the risk estimates is large, no scientific consensus on whether long-term residence in low-dose radiation areas causes health hazards for the time being [2,47,48]. Some implications can be found in the exposed group of Chernobyl accident. By 2008, among those exposed to radiation from the Chernobyl accident, a total of 6848 contracted thyroid cancer, all of whom were under the age of 18 in 1986 [37]. In contrast, no radiation-related thyroid cancer patients had been exposed to radiation as adults. As it is quite difficult to exclude other potential contributing factors, no clear correlation has been drawn between nuclear radiation exposure and the probability of contracting birth defects and genetic defects [49]. Apart from causing radiation-related diseases, radioactive contamination also has indirect negative effects on human health. According to statistics, the average life expectancy of individuals residing in disaster-stricken areas of Chernobyl was 65 years overall and only 59 years for males in 2003, which is far below the average life expectancy in other areas of Europe (76.0 for males and 81.7 for females in 2003) [49]. This is mainly a consequence of lifestyle-related illnesses, *i.e.*, cardiovascular disease, rather than diseases directly related to nuclear radiation, as living in radiation-contaminated areas is likely to reinforce anxiety, depression and other negative emotions, which in turn promotes intemperate lifestyle choices (*i.e.*, alcohol and tobacco abuse). Additionally, poverty-related malnutrition related to living in radiation-contaminated areas further increases the probability of early death [50,51]. 

In taking into account factors such as the quantity of contaminant discharge, the size of the contaminated area, and radiation levels suffered by affected populations, the Fukushima accident was far less severe than the Chernobyl nuclear accident [2,18,38]. However, most residents, and especially parents with children under the age of 18, are still deeply concerned about the long-term health effects of radiation in Fukushima [9,52], and hence they are not comfortable living in this area. As Minamisoma is an agriculturally oriented region, most residents used to work outside on farmland. Since the accident, residents have had to remain indoors to minimize their exposure to radiation, altering their lifestyle from one of active farming to one of inactivity and confinement [5]. The situation is even worse for school-aged children, as they can hardly play outside, and this may have adverse effects on their physical and mental development. According to the statistical yearbook for Minamisoma, obesity levels among fourth-grade children in 2012 had increased to two to three times the levels recorded in 2010 [12]. These trends had been largely consistent prior to the Fukushima accident. In addition to external radiation, the consumption of contaminated food or water could be a major source of chronic internal radiation exposure [19]. After the Fukushima accident, various concentration levels of ^137^Cs, ^134^Cs and ^131^I were identified in rice, vegetables, fruits, wild plants, milk, beef and seafood products produced in Fukushima [1]. Due to the extensive efforts in the food-monitoring program in Japan, the intake and internal exposure of the local residents is relatively low [18,53,54]. However, families residing close to the FDNPP still believe that they should avoid internal radiation by purchasing food from other areas at higher costs. In 2013, a survey on families with children residing in Minamisoma showed that local families imported 51% of their vegetable and fruit supply, 61% of their meat supply and 66% of their bottled mineral water supply (all the ratios are production value-based) from other areas of Japan and overseas [55]. In addition, the production value-based food self-sufficiency rate of Fukushima prefecture is 111% in 2008 [56]. As many parents do not wish to raise their children in a community without outdoor activities and with few food choices, most have not chosen to return. 

Delays in decontamination have also affected voluntary return trends. A survey on the repatriation intentions of evacuees now residing outside of Minamisoma shows that most respondents regard the effective control of radiation as a pre-condition for return [6]. However, the decontamination process has been delayed due to labor shortages and lack of effective decontamination technology [55]. In addition, Consent from landowners to dispose of waste around Minamisoma for the purposes of radiation decontamination has been difficult to obtain [23]. Additionally, decontamination can remove only 32%–51% of all radionuclides [22,23], as some residue is impossible to remove through existing means. In forests and other natural environments especially, decontamination efforts are highly complex and expensive [40,57,58]. Local meteorological conditions also affect contamination levels. Decontaminated or low radiation areas may, for instance, become high-risk areas after the occurrence of rain or a typhoon [2]. This uncertainty may causes residents to believe that long-term residence in contaminated areas poses health hazards [52]. Health effects from nuclear radiation have a minimum three-year latency period, and health risks to seniors are far less than those to children and youth [59]. Given difficulties that they face when adapting to new environments, evacuation to other areas may be more detrimental to the health of elderly individuals than remaining exposed to radiation [60]. As a result, seniors prefer to stay while young people are more willing to move to other areas for the sake of their own health and the health of their children.

### 4.2. Psychological Resilience

Disasters often have more enduring psychological effects than physiological effects on affected populations [37,51,61,62,63]. In comparing 16 disaster cases, S. Powell found that those who had suffered from the Chernobyl accident exhibit higher levels of physiological trauma and weaker psychological resilience than those who had suffered from other disasters [64]. Because the population is living in constant fear of radiation, the evacuees of the Fukushima accident exhibit the same degree of psychological trauma as those of the Chernobyl accident [2,52,61]. Compared to residents of other areas affected by the Great Eastern Japan Earthquake, residents of Fukushima are more psychologically vulnerable than residents of Miyagi-ken and Iwate-ken who suffered from the earthquake and tsunami only. Statistics from the Police Agency of Japan for August of 2011 suggest that, compared to the same period in 2010, suicide rates in Iwate-ken and Miyagi-ken had decreased by 19.4% and 18.4%, respectively, while the suicide rate in Fukushima increased by 7.5% [65]. These mental states prevail among forced and voluntary evacuees and workers residing in the FDNPP [37,66]. Given the persistence of radiation contamination, this mental state among victims is expected to endure for a long period of time [2]. A local resident of Minamisoma described this feeling in the following way: “I want to escape as far away as possible because the sight of this place (the area of Minamisoma where the evacuation advisory has been lifted) breaks my heart.” [55] Such painful memories may generate fear. Many evacuees living outside of the city do not possess the courage to resettle in Minamisoma.

Long-term symptoms of anxiety, depression and stress have had a greater effect on residents of nuclear-contaminated areas than direct effects of radiation [50]. Studies have shown that anxiety levels among Chernobyl accident victims are twice as high as those of a control group, and the former also exhibits three to four times more unexplained physical symptoms. Survivors feel weak, helpless and hopeless about the future rather than joyful at being alive [49]. A survey of post-disaster residents in Fukushima conducted by Tateno and Yokoyama showed that parents exhibit severe anxiety levels. The closer parents lived to the FDNPP, the higher the degree of anxiety found [52]. As the first nuclear accident occurred during the Internet era, the spread of troublesome unofficial information fosters trepidation and anxiety. Unlike scientists, ordinary citizens are more inclined to believe rumors and conspiracy theories related to radiation [67]. NHK, which is one of the biggest mass media in Japan, reported that 18 child thyroid cancer cases had been found in Japan in August 2013 [68]. Although no conclusive evidence existed to prove that these cases were related to the Fukushima accident, such reports have increased resident fears of nuclear radiation.

Public distrust of governmental bodies has affected the repatriation of young people. Affected residents’ concerns derive mainly from distrust in the government and inconclusive scientific information [52]. This anxiety in turn aggravates affected residents suspicions [2]. As a result of inadequate pre-disaster precautions and ineffective post-disaster protocols, the Japanese government and TEPCO have been criticized frequently and harshly by the public [69]. The inconsistent distribution of disaster-related information also causes the public to doubt the integrity of the government and of state-funded scientific institutions [16,52]. The fear of radiation also takes root in the fact that the monitoring of radionuclides has focused on some contaminants and has not covered the entire spectrum of emitted radionuclides yet [70]. Many people believe that “there is much more out there than what has been measured”. Nuclear radiation monitoring reports released by the Japanese government and by state-funded scientific research institutions are not fully trusted by the public [47]. The degree of distrust has not declined over time. After the NHRHQ lifted evacuation advisories on certain areas, the public still doubted the safety of these areas (including low dose areas and areas where decontamination has been achieved) despite the fact that these areas are declared suitable for safe return and long-term residence by the government.

Some researchers have found that the degree to which evacuees are tied to their home communities also affects their willingness to return [71,72,73]. Individuals who have lived in the area for generations or for an extended period of time tend to exhibit more attachment to their home communities and thus a higher willingness to return. Consequently, individuals prefer to remain in their home communities through great hardships rather than move to other areas [72,73]. In an interview with Fukushima residents, a female respondent over 80 years of age optimistically stated that she is not concerned about the hazards of nuclear radiation and that she intends to remain in the community that has been passed down from her ancestors. A male respondent of approximately 70 years of age also reported his intention to remain in the area to continue raising cattle despite the fact that nuclear radiation hazards related to such activities are significant [60]. This nostalgia for the native land and for existing ways of life causes senior residents to ignore hazards associated with nuclear radiation and to remain willing to reside in the home community. As young people are more willing to adapt to a new lifestyle, these individuals are more inclined to migrate.

### 4.3. Economic Fluctuation

Numerous economists have analyzed the economic factors that influence migration. In early research, wage differences, geographic distance, job market conditions (calculated based on unemployment and employment rates) and family traditions were identified as major economic factors that affect migration patterns [74,75,76]. In subsequent studies that examined the willingness to return among Hurricane Katrina evacuees, researchers found that economic conditions in disaster-stricken areas and costs associated with returning (including moving and house maintenance costs) also affect evacuee’s decisions. Job availability has also been identified as an important factor. Among individuals under the age of 30 with at least a bachelor degree, the willingness to return is low as such individuals can easily find jobs in other places. However, for those who originally held jobs in disaster-stricken areas, the willingness to return is higher [71]. 

Disasters have direct long-term effects on income, employment, industrial production and inflation levels in affected areas [34]. Compared to areas that had enjoyed higher degrees of economic vitality prior to a disaster, areas with fewer economic advantages are more likely to experience persisting and rapid population decline. A lack of leading industries in post-disaster areas restricts the return of residents to affected cities. For example, having been an underdeveloped city prior to Hurricane Katrina, New Orleans has only seen half of its evacuees return after two years recovery [77].

Major industries, i.e*.,* agriculture, fishing, retail and manufacturing industries [12], were all affected to varying degrees in Minamisoma following the Fukushima accident. As farmland was contaminated, the government restricted the sale of agricultural products from Fukushima [1,18,54]. Local farmers and the Minamisoma government have taken numerous measures to revive the agricultural sector, such as regularly measuring radiation levels in crops, degrees of Farmland decontamination, *etc.* [22]. However, the integrity of local agricultural products was damaged due to radiation contamination, and consequently these agricultural products, though deemed safe, are no longer marketable [18,78]. Due to the slow sale of crops, many peasant families no longer expect to experience an agricultural renaissance. A survey on the intentions of peasant families conducted in 2013 showed that 75% of peasant families in Odaka intended to either close or downsize their businesses [6]. To invigorate new industries, the Minamisoma government has proposed that peasant families collectively develop renewable energy resource industries. However, a consensus on this issue has not yet been reached among peasant families due to the large initial investment and high degree of land use redistribution involved, among other problems. The recovery of the manufacturing and retail industries is not proving optimistic either. Shelter advisories have forced enterprises to relocate from Minamisoma, and businesses that have resumed since the disaster are now facing human resource shortages and are running below full capacity. The decreasing number of businesses and decline in population after the disaster has consequently delayed the recovery of the retail industry. Although post-disaster financial aid for businesses is available (i.e., tax preferences, among other policies), Minamisoma does not attract external enterprises due to a lack provincial economic vitality [34]. This lack of traditional and new industries in turn hinders reconstruction and the return of residents to Minamisoma.

Meanwhile, the unavailability of long-term, stable jobs also discourages job seekers from migrating to Minamisoma. High-demand jobs available after the disaster largely pertain to reconstruction, such as building, civil engineering,* etc.* [34]. These positions are likely to be terminated after reconstruction is complete, and thus they are not appealing to job seekers. In addition, due to the impacts of radiation contamination, for families with minors, one parent must live in another area with the children while the other works in Minamisoma, and hence families are divided. These conditions render the city unattractive to job seekers.

## 5. Conclusions

Radiation contamination in areas affected by the Fukushima accident is becoming a chronic problem, as its effects may never be fully eliminated. Minamisoma, a predominantly agricultural area, has been devastated by the Fukushima accident. Although most areas in this city now show only low levels of radioactivity, evacuees, and especially those who are parents with children under the age of 18, prefer not to return. The affected area is experiencing population aging and decline, which in turn results in labor shortages, a declining consumer base and a vulnerable community environment overall. 

Why is it that so many evacuees do not intend to resettle? There are three possible reasons for this trend. First, former residents may be deterred by uncertainties surrounding the health effects of living in areas of low radioactivity. The majority of former residents are concerned about the direct and indirect effects of radiation on their health. In addition, decontamination delays have also affected perceptions of risk among evacuees now residing outside of the city, who largely believe that the city is still unsuitable for living. Second, post-disaster psychological disturbance and distrust of government information hinder immigration. Third, the affected area is unattractive to individuals residing outside of the area due to a scarcity of economic vitality and the occurrence of leading industry shutdown in Minamisoma.

Demographic problems and recovery processes are directly related. Uncertainties surrounding population return will cast a shadow on the recovery of this city, as many economic, cultural and social functions of cities are population-dependent. The long-term aftereffects that result consequently affect immigration patterns. An integrated plan is needed to bolster the affected cities’ ability to cope with the above-mentioned difficult circumstances.

## References

[1] Baba M. (2013). Fukushima Accident: What Happened?. Radiat. Meas..

[2] Steinhauser G., Brandl A., Johnson T.E. (2014). Comparison of the chernobyl and Fukushima nuclear accidents: A review of the environmental impacts. Sci. Total Environ..

[3] Suzuki I., Kaneko Y. (2013). Managing Fukushima NPS accidents: In particular focus on government crisis communication. Japan’s Disaster Governance.

[4] (2011). Final Report of The International Mission on Remediation of Large Contaminated Areas Off-Site the Fukushima Daiichi NPP.

[5] Ishikawa K., Kanazawa Y., Morimoto S., Takahashi T. (2014). Depopulation with rapid aging in Minamisoma city after the Fukushima daiichi nuclear power plant accident. J. Am. Vet. Med. Assoc..

[6] (2013). The Current Situation in Minamisoma City after the Nuclear Disaster.

[7] (2012). Main report Chapter 4. Overview of Damage from the Nuclear Power Plant Accident.

[8] (2013). Health Risk Assessment from the Nuclear Accident after the 2011 Great East Japan Earthquake and Tsunami Based on Preliminary Dose Estimation.

[9] Yamashita S., Suzuki S. (2013). Risk of thyroid cancer after the Fukushima nuclear power plant accident. Respir. Investig..

[10] International Atomic Energy Agency Fukushima Nuclear Accident Update Log. http://www.iaea.org/newscenter/news/2011/fukushima200511.html.

[11] Nuclear Regulation Authority Results of Airborne Monitoring by the Ministry of Education, Culture, Sports, Science and Technology and the U.S. Department of Energy. http://radioactivity.nsr.go.jp/en/contents/4000/3180/24/1304797_0506.pdf.

[12] (2013). Minamisoma Statistical Yearbook.

[13] Minamisoma Government The Geography of Minamisoma. http://www.city.minamisoma.lg.jp/index.cfm/8,1620,c,html/1620/04_3shou.pdf.

[14] Prime Minister of Japan and His Cabinet The Map of Evacuation Zone. http://www.kantei.go.jp/saigai/pdf/20140401gainenzu.pdf.

[15] Thielen H. (2012). The Fukushima daiichi nuclear accident—An overview. Health Phys..

[16] Onishi N., Fackler M. (2011). In nuclear crisis, crippling mistrust. New York Times.

[17] International Atomic Energy Agency Report of Japanese Government to the IAEA Ministerial Conference on Nuclear Safety—The Accident at TEPCO’s Fukushima Nuclear Power Stations. http://www.iaea.org/newscenter/focus/fukushima/japan-report/.

[18] Hamada N., Ogino H. (2012). Food safety regulations: What we learned from the Fukushima nuclear accident. J. Environ. Radioact..

[19] Sugimoto A., Gilmour S., Tsubokura M., Nomura S., Kami M., Oikawa T., Kanazawa Y., Shibuya K. (2014). Assessment of the risk of medium-term internal contamination in Minamisoma city, Fukushima, Japan, after the Fukushima Dai-Chi nuclear accident. Environ. Health Perspect..

[20] The Government of Japan, Ministry of Health Labor and Welfare Response to the Great East Japan Earthquake By the Ministry of Health, Labor and Welfare. http://www.mhlw.go.jp/english/topics/2011eq/index.html.

[21] Merz S., Steinhauser G., Hamada N. (2013). Anthropogenic radionuclides in Japanese food: Environmental and legal implications. Environ. Sci. Technol..

[22] Minamisoma Government Decontamination Plan in Minamisoma (The Third Version). http://www.city.minamisoma.lg.jp/index.cfm/10,16764,60,368,html.

[23] Ministry of Environment Progress on Off-Site Cleanup Efforts in Japan. https://josen.env.go.jp/en/pdf/progressseet_progress_on_cleanup_efforts.pdf?140425.

[24] Minamisoma Government The Current Situation of Recovery in Minamisoma City. http://www.city.minamisoma.lg.jp/index.cfm/10,5572,58,html.

[25] International Atomic Energy Agency IAEA Fukushima Daiichi Status Report. http://www.iaea.org/newscenter/focus/fukushima/statusreports/fukushima28_09_12.html.

[26] Matanle P. Ageing and Depopulation in Japan Understanding the Consequences for East and Southeast Asia By and Southeast Asia in the 21st Century. http://papers.ssrn.com/sol3/papers.cfm?abstract_id=2406498.

[27] Barusch A.S. (2011). Disaster, vulnerability, and older adults: Toward a social work response. J. Gerontol. Soc. Work.

[28] Phifer J., Kaniasty K., Norris F. (1988). The impact of natural disaster on the health of older adults: A multiwave prospective study. J. health Soc. Behav..

[29] Brown L.A., Shumway-Cook A., Woollacott M.H. (1999). Attentional demands and postural recovery: The effects of aging. J. Gerontol. Series A, Biol. Sci. Med. Sci..

[30] Adams V., Kaufman S.R., van Hattum T., Moody S. (2011). Aging disaster: Mortality, vulnerability, and long-term recovery among Katrina survivors. Med. Anthropol..

[31] Yasumura S., Goto A., Yamazaki S., Reich M.R. (2013). Excess mortality among relocated institutionalized elderly after the Fukushima nuclear disaster. Public Health.

[32] Koeda S., Narita H., Tsushima H. (2012). A literature review of health problems among nuclear power disaster evacuees: Common conditions, treatment, and rehabilitation. Radiat. Emerg. Med..

[33] Ishikawa K. (2013). Long-Term evacuation after the nuclear accident in Fukushima: Different daily living under low-dose radioactive suffering. Nihon Ronen Igakkai zasshi..

[34] Higuchi Y., Inui T., Hosoi T., Takabe I., Kawakami A. (2012). The impact of the great east Japan earthquake on the labor market: Need to resolve the employment mismatch in the disaster-stricken areas. Jpn. Labor Rev..

[35] Cardis E., Hatch M. (2011). The Chernobyl accident—An epidemiological perspective. Clin. Oncol..

[36] Tokonami S., Hosoda M., Akiba S., Sorimachi A., Kashiwakura I., Balonov M. (2012). Thyroid doses for evacuees from the Fukushima nuclear accident. Sci. Rep..

[37] United Nations Scientific Committee on the Effects of Atomic Radiation (2008). Effects of Ionizing Radiation: Report to the General Assembly, with Scientific Annexes.

[38] United Nations Scientific Committee on the Effects of Atomic Radiation (2014). Sources, Effects and Risks of Ionizing Radiation UNSCEAR 2013 Report: Report to the General Assembly, with Scientific Annexes A: Levels and effects of Radiation Exposure Due to the Nuclear Accident after the 2011 Great East-Japan Earthquake and Tsunami.

[39] United Nations Scientific Committee on the Effects of Atomic Radiation (2014). Sources, Effects and Risks of Ionizing Radiation UNSCEAR 2013 Report: 39 Scientific Annexes B: Effects of Radiation Exposure of Children.

[40] Steinhauser G., Merz S., Hainz D., Sterba J.H. (2013). Artificial radioactivity in environmental media (air, rainwater, soil, vegetation) in Austria after the Fukushima nuclear accident. Environ. Sci. Pollut. Res..

[41] Minamisoma Government Individual Internal Radiation Dose Test Results. http://www.city.minamisoma.lg.jp/index.cfm/10,0,61,367,html.

[42] Steinhauser G., Schauer V., Shozugawa K. (2013). Concentration of strontium-90 at selected hot spots in Japan. PloS ONE.

[43] Povinec P., Hirose K., Aoyama M. (2012). Radiostrontium in the western North Pacific: Characteristics, behavior, and the Fukushima impact. Environ. Sci. Technol..

[44] Zheng J., Tagami K., Watanabe Y., Uchida S., Aono T., Ishii N., Yoshida S., Kubota Y., Fuma S., Ihara S. (2012). Isotopic evidence of plutonium release into the environment from the Fukushima DNPP accident. Sci. Rep..

[45] Schneider S., Walther C., Bister S. (2013). Plutonium release from Fukushima daiichi fosters the need for more detailed investigations. Sci. Rep..

[46] Zheng J., Tagami K., Uchida S. (2013). Release of plutonium isotopes into the environment from the Fukushima daiichi nuclear power plant accident: What is known and what needs to be known. Environ. Sci. Technol..

[47] Normile D. (2013). Insistence on gathering real data confirms low radiation exposures. Science.

[48] Hayano R.S., Tsubokura M., Miyazaki M., Satou H., Sato K., Masaki S., Sakuma Y. (2013). Internal Radiocesium Contamination of Adults and Children in Fukushima 7 to 20 Months after the Fukushima NPP Accident as Measured by Extensive Whole-Body-Counter Surveys. Proc. Jpn. Acad. Ser. B, Phys. Boil. Sci..

[49] Kinley D., International Atomic Energy Agency (2006). Chernobyl’s Legacy: Health, Environmental and Socio-Economic Impacts and Recommendations to the Governments of Belarus, the Russian Federation and Ukraine.

[50] Stephan V. (2005). Chernobyl: Poverty and stress pose ‘bigger threat’ than radiation. Nature.

[51] Adams R.E., Guey L.T., Gluzman S.F., Bromet E.J. (2011). Psychological well-being and risk perceptions of mothers in Kyiv, Ukraine, 19 years after the Chornobyl disaster. Int. J. Soc. Psychiatry.

[52] Tateno S., Yokoyama H.M. (2013). Article public anxiety, trust, and the role of Mediators in communicating risk of exposure to low dose radiation after the Fukushima daiichi nuclear plant explosion. J. Sci. Commun..

[53] Harada K.H., Fujii Y., Adachi A., Tsukidate A., Asai F., Koizumi A. (2013). Dietary intake of radiocesium in adult residents in Fukushima prefecture and neighboring regions after the Fukushima nuclear power plant accident: 24-H food-duplicate survey in December 2011. Environ. Sci. Technol..

[54] Hamada N., Ogino H., Fujimichi Y. (2012). Safety regulations of food and water implemented in the first year following the Fukushima nuclear accident. J. Radiat. Res..

[55] Environmental Counselors Union The Current Situation of Recovery in Minamisoma. http://webcache.googleusercontent.com/search?q=cache:0jXvOqWizIYJ:fec.jyoukamachi.com/report/2013/2013_report_1.pdf+&cd=1&hl=zh-CN&ct=clnk&gl=jp.

[56] Ministry of Agriculture Forestry and Fisheries, Japan The Trend of Self-Sufficiency Rate and National Consciousness. http://www.maff.go.jp/j/wpaper/w_maff/h22/pdf/z_1_1_2.pdf.

[57] Pröhl G., Ehlken S., Fiedler I., Kirchner G., Klemt E., Zibold G. (2006). Ecological half-lives of ^90^Sr and ^137^Cs in terrestrial and aquatic ecosystems. J. Environ. Radioact..

[58] Yasutaka T., Naito W., Nakanishi J. (2013). Cost and effectiveness of decontamination strategies in radiation contaminated areas in Fukushima in regard to external radiation dose. PloS ONE.

[59] The International Commission on Radiological Protection (2007). The 2007 Recommendations of the International Commission on Radiological Protection.

[60] Sugimoto A., Krull S., Nomura S., Morita T., Tsubokura M. (2012). The voice of the most vulnerable: Lessons from the nuclear crisis in Fukushima, Japan. Bull. World Health Organ..

[61] Von Hippel F.N. (2011). The radiological and psychological consequences of the Fukushima daiichi accident. Bull. At. Sci..

[62] Norris F.H., Friedman M.J., Watson P.J., Byrne C.M., Diaz E., Kaniasty K. (2002). 60,000 disaster victims speak: Part I. An empirical review of the empirical literature, 1981–2001. Psychiatry.

[63] Chandra A., Acosta J.D. (2010). Disaster recovery also involves human recovery. JAMA : J. Am. Med. Assoc..

[64] Powell S., Plouffe L., Gorr P. (2009). When ageing and disasters collide: Lessons from 16 international case studies. Radiat. Prot. Dosim..

[65] JA Kyosai Research Institute The Suicide Caused by Earthquake: Direct Death and Indirect Death. http://www.jkri.or.jp/PDF/2011/Rep117hamada.pdf.

[66] Brumfiel G. (2013). Fukushima: Fallout of fear. Nature.

[67] Slovic P. (1996). Perception of risk from radiation. Radiat. Prot. Dosim..

[68] NHK Thyroid Cancer: Found in 18 Fukushima Children. http://www.youtube.com/watch?v=23dOVb76uzc.

[69] Leflar R., Hirata A. (2012). Human flotsam, legal fallout: Japan’s tsunami and nuclear meltdown. J. Environ. Law Litig..

[70] Steinhauser G. (2014). Fukushimas forgotten radionuclides: A review of the understudied radioactive emissions. Environ. Sci. Technol..

[71] Landry C.E., Bin O., Hindsley P., Whitehead J.C., Wilson K. (2007). Going home: Evacuation-Migration decisions of hurricane Katrina survivors. South. Econ. J..

[72] Chamlee-Wright E., Storr V.H. (2009). “There’s no place like new Orleans”: Sense of place and community recovery in the ninth ward after hurricane Katrina. J. Urban. Aff..

[73] Falk W.W., Hunt M.O., Hunt L.L. (2006). Hurricane Katrina and new Orleanians’ sense of place: Return and reconstitution or “Gone with the Wind”?. Du Bois Rev.: Soc. Sci. Res. Race.

[74] Graves P.E. (1980). Migration and climate. J. Reg. Sci..

[75] Walker R., Ellis M., Barff R. (1992). Linked migration systems: Immigration and internal labor flows in the United States. Econ. Geogr..

[76] Greenwood M.J. (1985). Human migration: Theory, models, and empirical studies. J. Reg. Sci..

[77] Vigdor J. (2008). The economic aftermath of hurricane Katrina. J. Econ. Perspect..

[78] Nakanishi T.M., Keitaro T. (2013). Agricultural Implications of the Fukushima Nuclear Accident.

